# Non-typeable *Haemophilus influenzae*-associated early pregnancy loss: an emerging neonatal and maternal pathogen

**DOI:** 10.1007/s15010-019-01359-6

**Published:** 2019-09-23

**Authors:** Muge Cevik, Olga L. Moncayo-Nieto, Margaret J. Evans

**Affiliations:** 1 Division of Infection and Global Health Research, School of Medicine, University of St Andrews, Fife, KY16 9TF UK; 2 Regional Infectious Diseases Unit, Western General Hospital, NHS Lothian, Edinburgh, UK; 3 Department of Laboratory Medicine, Royal Infirmary of Edinburgh, NHS Lothian, Edinburgh, UK; 4 School of Medicine, University of Edinburgh, Edinburgh, UK; 5 Department of Histopathology, Royal Infirmary of Edinburgh, NHS Lothian, Edinburgh, UK; 6 Centre for Comparative Pathology, University of Edinburgh, Edinburgh, UK

**Keywords:** Septic abortion, Pregnancy loss, Non-typeable *Haemophilus influenzae*, Invasive infections, Emerging pathogen, Maternal sepsis

## Abstract

**Objectives:**

There is increasing evidence indicating an association between invasive non-typeable* Haemophilus influenzae* (NTHi) infection in pregnancy and early pregnancy loss. As the diagnosis relies on microbiological investigation of post-mortem placental and foetal samples, a significant proportion of NTHi-related pregnancy loss remains unrecognised. To better characterise NTHi in septic abortion, we report NTHi cases associated with early pregnancy loss.

**Methods:**

We reviewed all post-mortems at <24 weeks gestation with histologically proven acute chorioamnionitis on placental histology and enrolled cases with at least one matched foetal and placental sample culture positive for NTHi. The study was approved by the NHS Lothian Caldicott Guardian.

**Results:**

In our cohort, invasive NTHi has accounted for 20% of infections associated with early pregnancy loss prior to 24 weeks gestation. All patients were young and healthy pregnant women at < 20 weeks' gestation who presented with abdominal pain, PV bleed /discharge and were septic at the time of presentation. One patient with previous history of miscarriage who presented with cervical incompetence had more severe pathology suggestive of early intrauterine pneumonia.

**Conclusion:**

The burden of invasive NTHi disease in early pregnancy loss is likely to be much larger than currently recognised. NTHi should be considered in pregnant women presenting with abdominal pain and PV bleed/discharge in whom clinical signs of sepsis are present. Active surveillance should be considered in this patient group including septic abortion to capture the true prevalence of this emerging pathogen to inform preventative and therapeutic approaches.

## Introduction

Non-typeable *Haemophilus influenzae* (NTHi) often causes upper respiratory tract infections and although rare, carries a potential risk of invasive disease especially in at-risk populations such as the elderly, children and neonates [1, 2]. Since the introduction of *H. Influenzae* vaccine, there has been a dramatic decline in mortality and morbidity associated with invasive disease caused by serotype b [3]. Subsequently, infections caused by NTHi have steadily increased over the past 20 years, now accounting for the majority of invasive disease [4, 5].

There is accumulating evidence indicating a strong association between invasive NTHi infection during pregnancy and early pregnancy loss as well as invasive neonatal disease [6, 7]. As the diagnosis relies on microbiological investigation of post-mortem placental and foetal samples, a significant proportion of NTHi-related pregnancy loss remains undiagnosed. Given the limited number of cases reported to date, the descriptive characteristics of patients and clinical presentation, potential mechanisms of spread and the optimum diagnosis and treatment are unexplored. To better characterise NTHi in septic abortion, we report early pregnancy loss cases prior to 24 weeks’ gestation associated with NTHi providing detailed information on the paired mother–foetal samples submitted for microbiological investigations (phenotypic and genotypic identification) and histopathology examination.

## Methods

A retrospective study was conducted in two different hospitals, in Edinburgh, Scotland from 1st of January 2017 to 31st of July 2018. During this period, all post-mortems at < 24 weeks gestation with histologically proven acute chorioamnionitis on placental histology were considered. We included cases of miscarriage defined as early pregnancy loss prior to 24 weeks in this study as according to the Stillbirth Act 1992, a foetus is considered viable after 24-week gestation. We enrolled patients with at least one matched foetal and placental sample culture positive for *H. influenzae*. Demographics, presenting symptoms and clinical data, including antimicrobial therapy and clinical outcome were extracted from the dedicated neonatal electronic medical records. All patient samples submitted for further evaluation have been identified, and detailed microbiological data were retrieved from the digital laboratory database and pathology data were obtained from the histopathology department on a case-to-case basis. The study was approved by the NHS Lothian Caldicott Guardian.

## Results

Thirty-three cases of histology confirmed acute chorioamnionitis associated with pregnancy loss prior to 24-week gestation were identified. In total, samples from 15 out of 33 cases were sent for culture. Out of the 15 culture positive cases, we identified three cases that were positive for* H. influenzae* with at least one matched foetal sample and placental sample. Maternal characteristics of these 3 cases included are presented in Table 1. The median age was 33 years (range 25–35), and all patients were at < 20 weeks’ gestation at the time of pregnancy loss. None had significant comorbidities including cervical treatment or surgery, but one had a previous history of miscarriage which had histological evidence of acute chorioamnionitis.

**Table 1:** Demographics and clinical presentation

	Patient 1	Patient 2	Patient 3
Age	35	22	33
PV bleed^a^	Yes	Yes	Yes
PV discharge	Yes	Yes	Yes
Abdominal pain	Yes	Yes	Yes
Fever	Yes	Yes	Yes
Gestation	14 weeks	14 weeks	20 weeks
Para	1	0	1
Previous miscarriage	No	No	Yes
History of chorioamnionitis	No	No	Yes
Funnelling of cervix	No	Yes	Yes
PROM^a^	No	No	Yes

^a^*PV* Per-vaginal, *PROM* premature rupture of membranes

Main symptoms at presentation were acute and severe cramping abdominal pain (100%), per vaginal (PV) bleeding (100%), PV discharge (100%) and all subsequently developed fever (100%). At the time of presentation, one case experienced premature rupture of membranes (PROM) and two out of three patients had clinical signs of sepsis requiring fluid resuscitation and immediate antimicrobials. One patient developed sepsis shortly after delivery of the placenta. The median duration of symptoms was 48 h [range, 24–72 h]. Median white blood cells count was 17.0 10^9^/L (range, 15–21.2) with neutrophilia, median CRP level was 167 mg/L (range, 28–185). On speculum examination, all patients had blood stained PV discharge, and cervix was noted to be closed. Transvaginal scanning revealed the evidence of cervical funnelling in two patients.

Although all patients had clinical signs of sepsis at the time of presentation, *H. influenzae* was cultured from blood cultures and high vaginal swabs (HVS) in two maternal cases and one patient had negative microbiology. Further examination of medical notes implied that this patient was administered antibiotics swiftly after presentation which might have influenced the culture results. Additional infection screen in all maternal cases including CMV, EBV, parvovirus B19, rubella and toxoplasmosis serology, midstream urine culture, was negative, and all patients had normal Chest XR. Treatment included β-lactam antimicrobials for all patients with a median duration of seven days (range, 7–10) and supportive management which culminated in an examination under anaesthesia for evacuation of retained placenta in two cases.

The diagnosis of *H. influenzae* was obtained both from the placental sample and either from foetal blood or foetal tissue culture. In post-mortem samples sent for microbiological examination, *H. influenzae* isolates identified were β-lactamase negative, penicillin resistant and ampicillin susceptible. All *H. Influenzae* isolates had further phenotypic characterisation by PCR-based capsular typing confirming NTHi. Genotypic characterization by multilocus sequence typing has identified sequence types as ST408 and ST145.

**Fig. 1:**
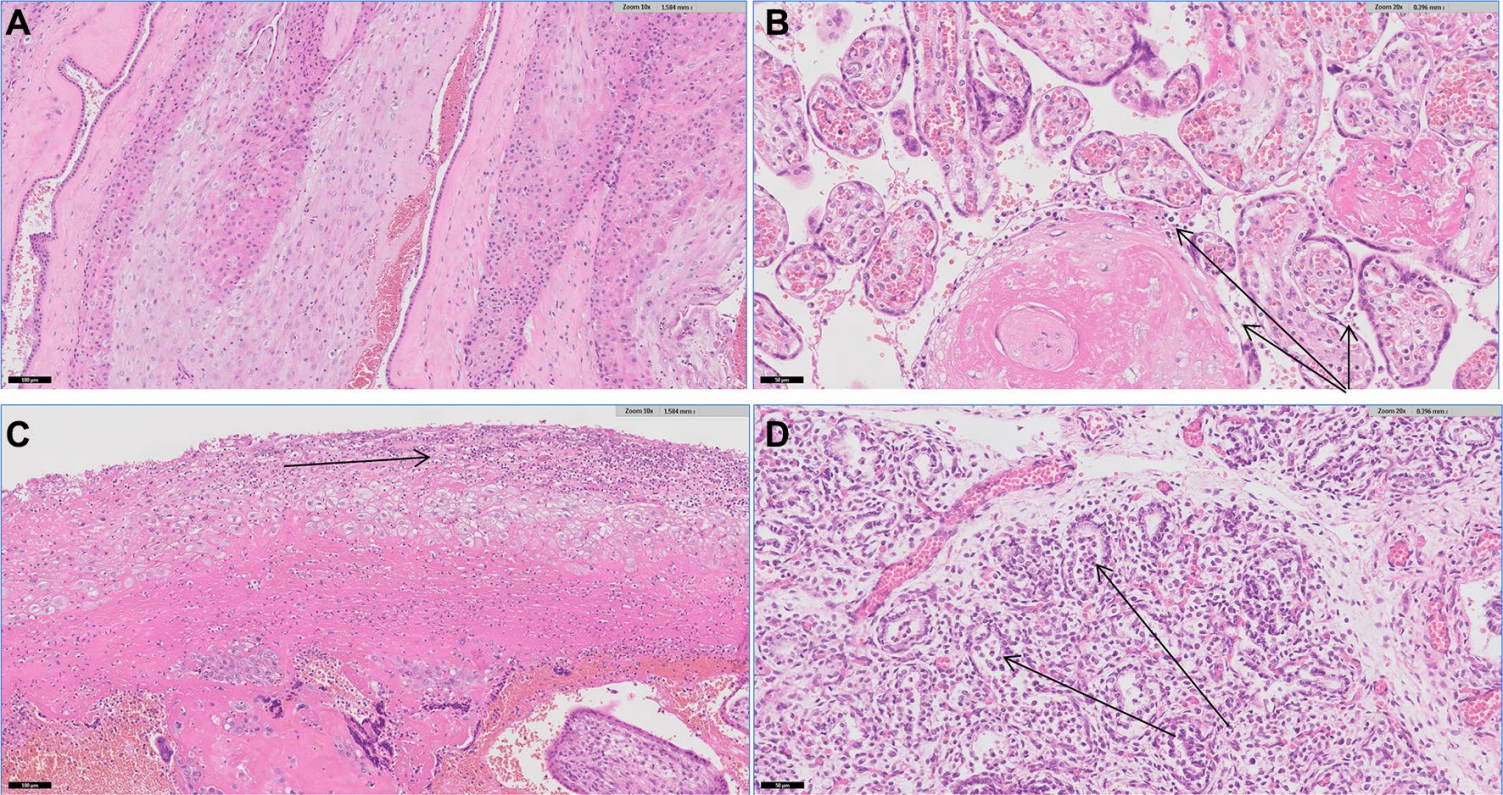
Histopathology images of normal amnion and abnormal amnion from the cases: **a** normal amnion with no acute chorioamnionitis. **b** Scanning H and E micrograph showing intervillositis (arrows: acute inflammatory cells within the intervillous space). **c** Scanning H and E micrograph showing acute necrotising chorioamnionitis (Arrow: loss of amnion and dense inflammatory cell infiltrate). **d** Scanning micrograph showing acute inflammatory cell infiltrate within alveoli

Histopathological evaluation of all placenta samples demonstrated an acute necrotising chorioamnionitis with no evidence of vasculitis or funisitis. Samples from patient 3 showed an acute intervillositis with evidence of an acute inflammatory cell infiltrate within the putative alveoli suggestive of early congenital or intrauterine pneumonia (Fig. 1).

## Discussion

We report three cases of confirmed NTHi associated with early pregnancy loss in paired maternal and foetal samples. The main findings were: (1) in our cohort, invasive NTHi has accounted for 20% of infections associated with early pregnancy loss prior to 24-weeks’ gestation; (2) NTHi should be considered in young and healthy pregnant women presenting with abdominal pain, PV bleed/discharge and sepsis.

*H. influenzae* is a treatable cause of adverse pregnancy outcomes, yet the fastidious nature of this Gram-negative coccobacillus makes the recognition and the diagnosis challenging. Population-based studies have proposed increased susceptibility to invasive *H. influenzae* infections during pregnancy. According to a surveillance data from England, pregnant women had 17-fold increased risk of invasive NTHi compared with non-pregnant women [7]. Among pregnant women with invasive *H. influenzae*, the incidence of pregnancy loss was 61%, 20% of which were lost prior to 25 weeks’ gestation [7]. In our cohort, invasive NTHi has accounted for 20% (3/15) of infections associated with early pregnancy loss. However, over half of the post-mortem samples were not sent for culture. This supports the available evidence that intrapartum sepsis due to NTHi which has a specific affinity to the genital tract is becoming more prevalent in this population [5]. While enhanced management of intrapartum sepsis promoted by international guidelines had a significant impact on the maternal and neonatal mortality outcomes over the recent years, intrapartum sepsis remains a common yet potentially preventable cause of adverse pregnancy outcomes [8]. This emphasises the necessity for a preventative approach, such as early detection of NTHi and advanced case finding in early pregnancy.

Ascending vaginal or cervical infection is often the suspected route of transmission. In the present case series, two out of three patients had cultured NTHi on HVS samples which supports the hypothesis that NTH- related foetal loss likely results from ascending infection. Although NTHi genital carriage rates have been documented to be lower compared to Group-B streptococcus, (1.8/1000 pregnant women), higher rates were reported in women with PROM [9, 10]. While histopathology from all samples demonstrated an acute necrotising chorioamnionitis with no evidence of vasculitis or funisitis in all cases, one of the patients in this case series had more severe pathology suggestive of early congenital or intrauterine pneumonia. This patient had history of miscarriage with evidence of acute chorioamnionitis and presented at 20 weeks’ gestation with cervical incompetence subsequently experiencing PROM. This emphasise the need to consider NTHi among pregnant women who previously had cervical surgery or previous miscarriage presenting with abdominal pain, PV bleed/discharge and cervical incompetence.

Although there is growing evidence of resistance in NTHi due to β-lactamase-positive, ampicillin-resistant strains associated with septic abortion [5, 11], in the present case series, all isolates were β-lactamase negative, penicillin resistant and ampicillin susceptible and all patients were successfully treated with β-lactam antimicrobials. This adds additional importance to this emerging pathogen and calls for appropriate detection to inform diagnostic and therapeutic approaches in early pregnancy. The primary step of microbiological and histopathological sampling is critical to generating data to inform clinical management of infections in early pregnancy. This highlights the value in implementing standardised surveillance protocols and typing methods to monitor this emerging pathogen.

We acknowledge the limitations of this case series. Firstly, due to its retrospective nature, some relevant information might have been missed during data collection. Although our case series is unique to incorporate paired maternal and foetal samples, sample size remains limited. Finally, this case series may not have identified the true burden of NTHi as cases of miscarriage, septic abortions, and stillbirths rarely undergo a post-mortem examination.

In conclusion, accumulating evidence suggests that the burden of invasive NTHi disease in early pregnancy loss is likely to be larger than currently recognised [5, 7]. NTHi should be considered in pregnant women in the early pregnancy presenting with abdominal pain and PV bleed/discharge in whom clinical signs of sepsis are present. Screening for NTHi should be considered for pregnant women who previously had cervical surgery, miscarriage or presenting with cervical incompetence. The clinicians are urged to be vigilant regarding this emerging pathogen and every effort should be undertaken to detect NTHi in all cases including septic abortion to capture the true prevalence of this pathogen especially in the early pregnancy prior to 24 weeks’ gestation to inform preventative and therapeutic approaches.
